# Brain sensory network activity underlies reduced nociceptive initiated and nociplastic pain via acupuncture in fibromyalgia

**DOI:** 10.1038/s43856-025-01280-0

**Published:** 2026-01-10

**Authors:** Apeksha Sridhar, Ishtiaq Mawla, Eric Ichesco, Brock Pluimer, Steven E. Harte, Robert Edwards, Vitaly Napadow, Richard E. Harris

**Affiliations:** 1 Susan Samueli Integrative Health Institute, School of Medicine, University of California at Irvine, Irvine, CA USA; 2 Department of Anesthesiology and Perioperative Care, School of Medicine, University of California at Irvine, Irvine, CA USA; 3 Chronic Pain and Fatigue Research Center, Department of Anesthesiology, University of Michigan Medical School, Ann Arbor, MI USA; 4 Department of Anesthesiology, Critical Care and Pain Medicine, Brigham and Women’s Hospital, Harvard Medical School, Boston, MA USA; 5 Martinos Center for Biomedical Imaging, Massachusetts General Hospital, Harvard Medical School, Boston, MA USA; 6 Department of Physical Medicine and Rehabilitation, Spaulding Rehabilitation Hospital, Harvard Medical School, Boston, MA USA

**Keywords:** Chronic pain, Chronic pain, Insula

## Abstract

**Background:**

Chronic pain may involve both nociceptive pain driven by peripheral tissue damage and nociplastic pain reflecting central nervous system dysregulation, as in fibromyalgia. Electroacupuncture has been shown to modulate these pathways, but the underlying brain mechanisms remain unclear. This study investigated how electroacupuncture influences nociceptive-initiated and centrally maintained pain via changes in brain activation and functional connectivity.

**Methods:**

In this randomized controlled trial (NCT02064296), female adults with fibromyalgia received either electroacupuncture (n = 19) or sham treatment with inactive laser stimulation (n = 25) over four weeks. Changes in brain activation and connectivity during evoked pressure-pain stimulation were assessed using functional magnetic resonance imaging before and after treatment. Here, we present a secondary analysis of data from the trial. Clinical outcomes assessed include pressure-pain tolerance and widespread pain, and analyses tested whether brain measures mediated treatment-related effects.

**Results:**

Here we show that in the electroacupuncture group, reductions in widespread pain are associated with increases in pressure-pain tolerance. This relationship is mediated by greater activation of the primary somatosensory cortex and stronger connectivity between somatosensory and insular regions, consistent with a bottom-up mechanism linking peripheral nociceptive-initiated input to central nociplastic pain modulation. In contrast, the sham group shows reductions in widespread pain linked to decreased precuneus activity and precuneus–insula connectivity, consistent with a top-down process.

**Conclusions:**

Electroacupuncture and sham treatments engage distinct neural pathways to influence pain perception. These findings highlight that electroacupuncture modulates nociceptive-initiated and nociplastic pain through a bottom-up sensory pathway, whereas sham treatment engages top-down control. This mechanistic dissociation may inform patient selection and optimization of acupuncture-based therapies for chronic pain.

## Plain language summary

Fibromyalgia is a long-term condition that causes widespread pain, fatigue, and increased sensitivity. Pain in fibromyalgia can come from signals in the body (nociceptive pain) and from how the brain processes those signals (nociplastic pain). This study tested how electroacupuncture, a form of acupuncture that uses gentle electrical pulses, affects these pain pathways. Adults with fibromyalgia received either four weeks of electroacupuncture or a placebo-like treatment. Brain scans and pain tests were done before and after treatment. Electroacupuncture increased people’s tolerance to pressure pain and changed activity in sensory regions of the brain that detect and interpret pain. These brain changes were linked to reduced widespread pain, suggesting that pain relief after electroacupuncture begins in the body and then leads to changes in the brain. In contrast, the placebo treatment affected only brain pathways linked to nociplastic pain. Electroacupuncture may therefore benefit people with both nociceptive and nociplastic pain.

## Abstract (web summary)

Sridhar et al. examine how electroacupuncture modulates brain activity and connectivity in fibromyalgia using pre- and post-treatment fMRI. They show that electroacupuncture engages somatosensory–insula circuits to link nociceptive-initiated pain with reduced nociplastic widespread pain.

## Introduction

Chronic pain conditions are often challenging to treat because of their complex, multifaceted nature and variability among individuals^1^. The current understanding of this complexity is that pain arises from three primary processes: nociceptive, neuropathic, and nociplastic pain mechanisms^2–4^. Nociceptive pain arises from tissue damage or inflammation, which activates peripheral nociceptors, whereas neuropathic pain stems from nervous system injury^2–4^. In contrast, nociplastic pain is thought to result from central nervous system (CNS) dysfunction, where pain signals are amplified despite the absence of clear peripheral tissue damage, inflammation, nerve lesions or disease^2–4^. While these classifications offer a useful framework, many patients with chronic pain display overlapping characteristics of these mechanisms, with multiple pain processes coexisting. Disentangling these characteristics can be crucial for targeted treatments based on nociplastic-dominant versus nociceptive-dominant phenotypes. For instance, chronic pelvic pain often involves both nociceptive components, such as endometriosis or pelvic inflammatory disease, and nociplastic processes driven by central nervous system dysfunction. In women with chronic pelvic pain undergoing hysterectomy, those with greater preoperative markers of central sensitization were more likely to experience persistent postoperative pain, highlighting the limited efficacy of peripheral interventions in individuals with primarily nociplastic pain profiles^5^.

Fibromyalgia (FM) is an example of a chronic pain condition in which both peripherally driven (nociceptive) and central (nociplastic) mechanisms may contribute to chronic pain intensity^6^. FM is primarily characterized by widespread pain but is also associated with fatigue, sleep disturbances, and cognitive impairments^3^. Neuroimaging studies in FM have shown altered brain activity in pain-processing regions, including the insula, anterior cingulate cortex (ACC), and primary somatosensory cortex (S1), in the absence of external stimuli^7^ as well as in response to non-painful and non-somatic sensory input^8,9^, indicating a generalized hypersensitivity in central sensory processing systems. These findings suggest that chronic pain in FM may be perpetuated by abnormal central pain processing, commonly referred to as central sensitization, rather than ongoing peripheral injury^6,10^. Further supporting this, resting-state functional connectivity (FC) studies have shown increased connectivity between the default mode network (DMN) and salience network (SLN), particularly in the anterior insula, which correlates with heightened pain sensitivity and impaired pain regulation^11^. Additionally, altered neurochemistry in the insula has been observed in FM, including reduced levels of γ-aminobutyric acid (GABA)^12^ and elevated levels of glutamate^13^, suggesting impaired inhibitory modulation of pain that contributes to the amplification of widespread pain symptoms in FM.

Individuals with FM also exhibit heightened sensitivity (i.e., reduced pain thresholds) to nociceptive stimuli, such as pressure or heat, suggesting that increased sensitivity to nociceptive pain may also contribute to central nociplastic processes in some patients^14,15^. One of the first studies in FM showed that patients with FM exhibit increased brain activation in pain-processing regions, including the S1 and insula, in response to a peripheral nociceptive stimulus^14^. A potential contributor to this enhanced nociceptive sensitivity is peripheral small fibre neuropathy which has been reported in many FM patients and may play a role in altered sensory perception, although its causal link to FM symptoms remains uncertain^16,17^. While it is possible that nociceptive afference may be normal in FM and this is simply amplified in the CNS, another possibility is that a combination of both peripheral nociceptive and central nociplastic mechanisms may both contribute to the complex pain experience in some individuals with FM^2^. Indeed, we have proposed that both peripheral “bottom up” and central “top down” processes may contribute to pain in FM^2^.

Electroacupuncture (EA), a modern variation of traditional acupuncture that involves electrical stimulation delivered via acupuncture needles, has emerged as a promising non-pharmacological treatment for multiple pain disorders, including FM^18^. These clinical findings are supported by neuroimaging studies, which show that EA might target nociplastic pain by modulating activity in pain-processing regions, recalibrating brain connectivity in pain-facilitatory circuits^19–23^, and engaging descending pain inhibitory systems^24^. EA is also shown to enhance the release of endogenous opioids^25^ and modulate neurotransmitters such as endocannabinoids^26^, serotonin, dopamine, and GABA, which are also critical for pain modulation^27–29^. In addition to these central effects, EA alleviates nociceptive pain by activating mechanoreceptors in the skin and muscles, which stimulate afferent nerve fibers triggering spinal and supraspinal pain inhibitory pathways, reducing nociceptive input and inflammation^29^.

Although existing findings indicate some central effects of EA, they offer limited insight into the mechanistic pathways by which EA modulates different pain processes. Understanding how EA engages multiple pain mechanisms is essential for tailoring treatments to each patient’s unique pain profile. The lack of a clear framework for matching specific treatment characteristics to individual pain mechanisms likely contributes to variability in outcomes, with interventions succeeding in some patients but failing in others. To the best of our knowledge, our study is one of the first to bridge this critical gap, as we investigate the neural pathways through which EA targets both nociceptive and nociplastic pain mechanisms via somatosensory brain networks in FM. This is an essential first step toward disentangling overlapping pain mechanisms and developing individualized treatments for those with chronic nociplastic pain diagnoses.

In the present study, we show that EA engages distinct brain pathways to link nociceptive-initiated and nociplastic pain processes in FM. Specifically, EA links increased pain tolerance and reduced widespread pain, which is mediated by greater activation of the primary somatosensory cortex (S1) and stronger connectivity of S1 with the anterior insula. By contrast, the sham treatment engages different brain networks consistent with a top-down modulatory process. These findings suggest that EA may modulate pain through a bottom-up sensory mechanism and highlight its potential for patients with mixed pain profiles.

## Methods

### Overall protocol

This study was a single-site, blinded, sham-controlled, randomized, non-crossover longitudinal neuroimaging trial. It was pre-registered with ClinicalTrials.gov (NCT02064296) on December 11, 2013 and conducted at the University of Michigan, Ann Arbor, MI, between December 2014 and November 2019. The study received approval from the University of Michigan Medical Institutional Review Board, and all participants provided written informed consent in line with the Declaration of Helsinki.

The primary endpoint (Brief Pain Inventory pain severity) and secondary endpoints, including resting-state functional connectivity and anterior insula neurochemistry, have been published previously^21^. Exploratory neuroimaging findings from this dataset have also been reported^22,30^. However, this study newly reports relationships between brain activity and connectivity to evoked pain and resulting changes in nociceptive-initiated and nociplastic pain following EA.

### Participants

Participants diagnosed with FM were recruited for this study and met the 2011 FM Survey Criteria, self-reported symptoms for at least one year, pain on more than 50% of days, and a pain score of ≥ 4 on a 10 cm Visual Analog Scale (VAS). All participants were female and right-handed, as FM predominantly affects females and this design allows direct comparison with prior female FM cohorts. Exclusion criteria included having received acupuncture within the last 6 months, neurological disorders such as peripheral neuropathy, psychiatric conditions (e.g., schizophrenia, major depression with suicidal ideation), substance abuse, use of opioids or stimulant medications, and contraindications to MRI or electrostimulation. Further details on inclusion/exclusion criteria and medication usage are available in our previous publication^21^.

Participants were randomized into EA or mock laser (ML), a sham control mimicking acupuncture without somatosensory afference (described below). Of the 70 participants with clinical data, 26 were excluded due to missing either baseline or post-treatment evoked pain scans. No participants were excluded for excessive motion (see details below), resulting in a final fMRI sample of 44 participants (EA: 19, ML: 25). Power analysis using NeuroPower^31^ indicated that our total sample size of 44 subjects provides 82% power under Bonferroni correction (α = 0.05). All participants completed behavioral assessments, quantitative sensory testing (QST), as well as neuroimaging sessions at the University of Michigan. Clinical characteristics and participant demographics for both the full sample and the fMRI subsample are provided in Supplemental Tables 1 and 2.

### Experimental design

**Fig. 1:**
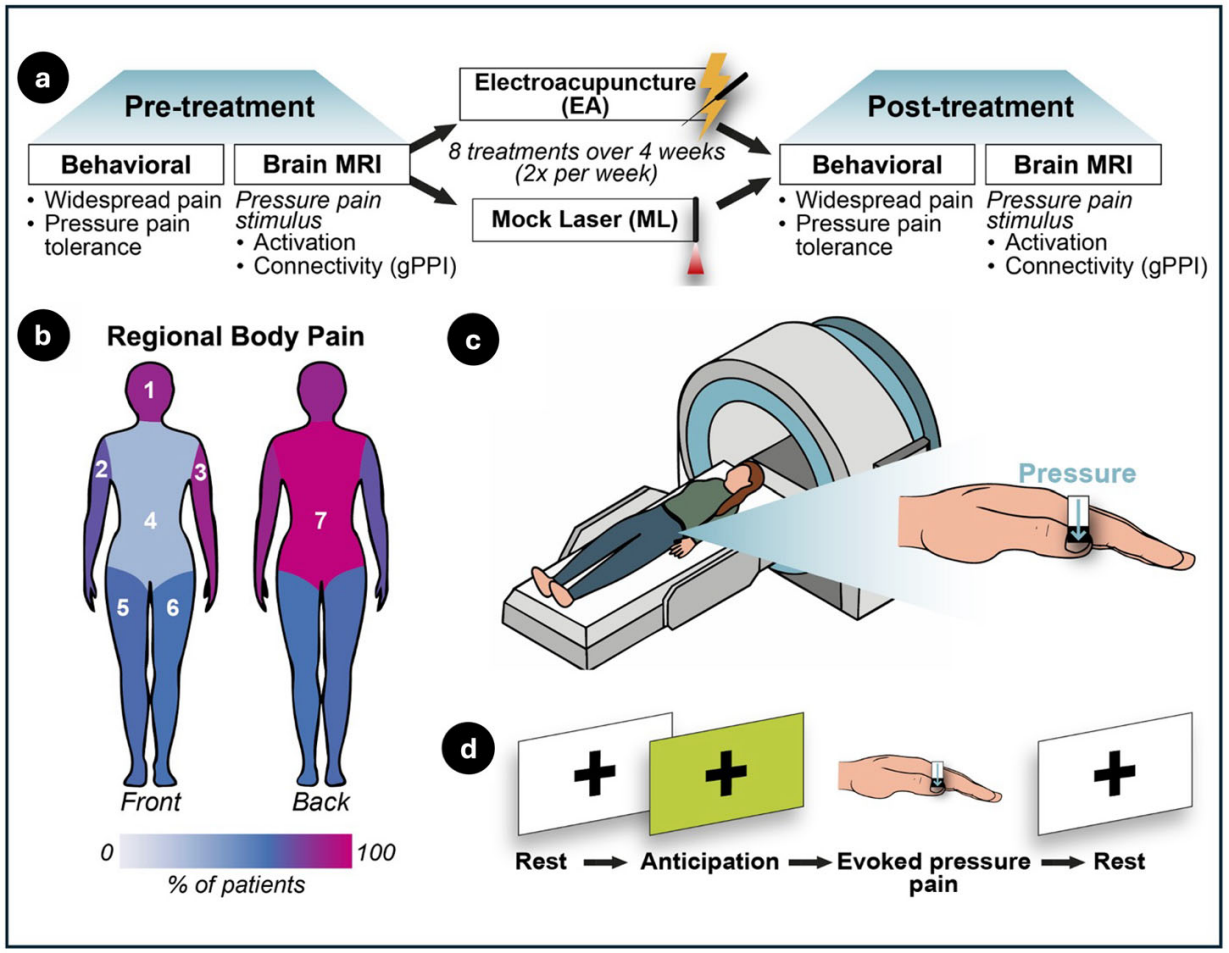
Experimental design and pain assessments. **a** Timeline of the study, including baseline assessments (left), treatment with electroacupuncture (EA) or control mock laser (ML) acupuncture (center), and post-treatment evaluations (right). **b** Seven body regions used to assess widespread nociplastic pain, with colors representing the percentage of participants reporting pain in each region for both groups combined prior to treatment. **c** Illustration of the nociceptive-initiated pressure-pain stimulus applied during functional magnetic resonance imaging (fMRI) sessions. **d** Block design used in fMRI scans, showing rest, anticipation, and evoked pressure-pain phases.

A full description of our study design is displayed in Fig. 6. In brief, participants first underwent a screening session to confirm eligibility and familiarize themselves with the study procedures. They were then randomized using a computer-generated randomization sequence to receive either EA or ML. The comparison between EA and ML was designed to isolate the effects of somatosensory stimulation: EA involves direct sensory input via needle insertion and electrical stimulation, whereas ML serves as a sensory-inactive control, enabling the examination of brain and behavioral responses specifically driven by afferent somatosensory signaling. Behavioral and MRI data were collected from participants before and after treatment (Fig. 1a).

QST included an assessment of pressure-pain tolerance (PPTol) during which pressure stimuli were applied to the left thumbnail bed using the Multimodal Ascending Sensory Test (MAST) system (Arbor Medical Innovations, Saline, MI)^30,32^. A series of discrete pressure stimuli were delivered in ascending order, starting at 0.25 kgf/cm^2^ and increasing in steps of 0.25–0.50 kgf/cm^2^ to a maximum of 10.0 kgf/cm^2^. Participants rated the magnitude of pain evoked at each pressure level on a digital 0–100 Numerical Rating Scale (NRS), anchored at 0 (“no pain”) and 100 (“worst pain imaginable”). Testing stopped when the maximum pressure was reached, or when participants provided a rating of ≥ 80/100 or indicated they were unwilling to continue testing. PPTol was defined as the last pressure recorded during testing before stopping. In addition, stimulus-response data from the entire ascending series were used to interpolate individualized P30 thresholds for each participant, defined as an approximation of the pressure intensity that evokes a rating of 30 on the NRS. P30 thresholds were used in subsequent magnetic resonance imaging (MRI) sessions.

The spatial distribution of pain across the body (widespread pain) was assessed using the FM Survey Criteria^33^. The 19 body sites from the FM Survey Criteria’s Widespread Pain Index subscale were grouped into seven regions: head, front, back, right arm, right leg, left arm, and left leg (Fig. 1b). This grouping was applied to provide a more clinically meaningful measure of widespreadness, reducing the likelihood of overestimating pain distribution by counting multiple adjacent sites within the same limb or region as separate occurrences. Changes in widespread pain were quantified by calculating the difference in the total number of body regions endorsed as painful prior to and following treatment (i.e., post-treatment values minus pre-treatment values).

To assess treatment-related changes in PPTol and widespread pain, we conducted paired-samples *t*-tests using SPSS (IBM Statistical Package for the Social Sciences, version 29.0.2.0) to determine whether each measure showed a significant change from pre- to post-treatment (at *p* < 0.05). To examine the relationship between PPTol and widespread pain, we performed Spearman correlation analyses in SPSS. In addition, Cocor was used to statistically compare the strength of correlations between groups.

Following each behavioral session, participants completed a functional MRI session. To reduce head motion, participants were positioned in their scanner with padding surrounding their heads in the head coil to reduce movement. The session included a block-design task involving alternating periods of rest, pressure pain applied to the left thumbnail bed, and a green cross visually displayed on a monitor indicating the anticipation of pressure stimulation (Figs. 1c, d). Pressure stimuli during the pain blocks were delivered in the scanner using an MRI-compatible device (IPC-1000 Thumb Stimulator; Arbor Medical Innovations, Saline, MI), which applied calibrated pressure through a handpiece connected via tubing routed through the MRI access port. Stimulus timing was precisely synchronized with the experimental protocol using E-Prime software. During the entire scan, participants were instructed to remain still with their eyes open, and to focus on the screen. Each block consisted of a 10 s P30 pressure stimulus on the left thumbnail bed, preceded by an anticipation cue (green cross, jittered 4–10 s) and followed by a rest period jittered 10–20 s (Supplemental Table 3). In addition to the block-design task, a resting-state scan (at the beginning of the scan session), proton magnetic resonance spectroscopy (^1^H-MRS) and other experimental scans were collected, including tests involving pressure on the calf; however, these scans were not included as they have been reported on previously^21,22^.

### Acupuncture treatment

Participants in the EA and ML groups underwent eight treatment sessions, administered twice weekly over four weeks. In the EA group, participants received electrical stimulation at three pairs of acupoints: right LI-11 (lateral elbow) to LI-4 (dorsum of hand), left GB-34 (lateral knee) to SP-6 (medial lower leg), and bilateral ST-36 (anterior lower leg)^21^. These acupoints were selected based on their clinical relevance to common FM symptoms, such as multisite pain, headaches, gastrointestinal dysfunction, disrupted sleep, and chronic fatigue^34^. A low-frequency EA device delivered electrical stimulation with pulse width, frequency, and intensity individualized to each participant, based on their sensory and pain thresholds. The electrostimulation targeted central pain pathways thought to modulate widespread pain. Sessions lasted 25 min. Additional details about the treatment protocol, including the specific parameters of electrostimulation, are provided previously^21^.

In the ML sham control group, participants were told they would receive acupoint stimulation using a laser device; however, the device was inactive and emitted no therapeutic laser energy. This intervention mimicked the appearance and procedure of real treatment while lacking any somatosensory stimulation, serving as a sham control for all EA analyses. The ML sham control (inactive laser) has been considered a credible placebo in prior methodological work^35^, and has been used as a sham control in RCTs with successful blinding of participants and practitioners^36,37^. This authorized deception was approved by the University of Michigan IRB. The ML device was positioned over the same acupoints as in the EA group. To ensure blinding and credibility, both groups wore blindfolds during treatment and received identical verbal instructions and treatment durations. Participants were fully debriefed after the study. Additionally, a Credibility Questionnaire was administered after the first and last treatment sessions to evaluate participants’ perceptions of the treatment’s validity, ensuring that any observed clinical or neuroimaging differences were not influenced by disparities in treatment credibility.

### MRI acquisition and preprocessing

Neuroimaging was conducted using a 3.0 T Philips Ingenia MRI scanner with a standard 32-channel head coil at the University of Michigan. High-resolution anatomical images covering the whole brain were acquired using a T1-weighted sequence (repetition time [TR] = 8.2 ms, echo time [TE] = 3.7 ms, matrix = 240 × 240, flip angle = 8°, field of view (FOV) = 256 mm, voxel size = 1 mm³, 172 slices), providing detailed structural information for subsequent coregistration with functional data. Functional MRI (fMRI) data were collected using a T2*-weighted echo-planar imaging (EPI) sequence to measure blood oxygenation level-dependent (BOLD) signals during task-based conditions. Each task fMRI scan had the following parameters: volumes = 102, TR = 2 s, TE = 30 ms, flip angle = 90°, image size = 2.75 × 2.75 mm^2^, matrix = 80 × 80, 38 axial slices, and slice thickness = 3.5 mm.

fMRI data were preprocessed using fMRIPrep^38^ (v23.2.0), which included skull stripping, motion correction via rigid body realignment, and nonlinear spatial normalization to MNI152 standard space. Slice timing correction was not applied because our block-design paradigm operates on a timescale much longer than slice-acquisition offsets, and prior work suggests that the benefits of STC are modest for block designs with TR ≈ 2 s^39–41^. The first 5 volumes of each functional run were discarded to allow for signal equilibration and reduce T1 stabilization effects. Functional images were then smoothed with a 6 mm FWHM Gaussian kernel using AFNI’s 3dBlurInMask to enhance signal-to-noise ratio^42^. Framewise displacement (FD) was calculated for each run, and volumes exceeding 0.5 mm FD were flagged for motion scrubbing^43,44^. Participants were excluded from analysis if more than 30% of volumes in a run exceeded the 0.5 mm FD threshold^45^; however, no participants in the present dataset met this exclusion criterion. Additionally, nuisance regressors, including six head motion parameters (translation and rotation) and CompCor components, were included to further denoise the data and remove physiological noise from the functional connectivity (FC) analysis.

### Statistics and reproducibility

This study included 44 female participants randomized to receive either EA (*n* = 19) or ML (*n* = 25). Normality of behavioral data was assessed with Shapiro–Wilk tests and homogeneity of variances with Levene’s test. When assumptions for parametric tests were not met, non-parametric alternatives were applied (e.g., Spearman correlations for associations between PPTol and widespread pain). Neuroimaging analyses employed second-level GLMs in SPM12 and CONN with voxel-wise thresholds at *p* < 0.001 (uncorrected) and cluster-level FWE correction at *p* < 0.05. Mediation models were tested with bias-corrected bootstrapping (5000 samples). Reproducibility was ensured by prespecified quality control (e.g., discarding 5 initial volumes, FD threshold of 0.5 mm, exclusion if >30% volumes exceeded threshold) and by treating each participant as an independent replicate.

### Brain activation analysis

First-level statistical analyses were conducted using Statistical Parametric Mapping (SPM12). A general linear model (GLM) was applied to model BOLD responses during the pressure-pain task conditions. The model included four conditions: rest (10–20 seconds), green fixation cross (anticipatory, no painful stimulation; 4–10 s), pressure-pain ramp (4 s), and pressure-pain plateau (6 s). The pain ramp condition specifically targeted the initial 4 s of the pain condition to capture early pain perception. This approach was based on evidence that modeling the onset (on-ramp) and offset (off-ramp) periods of pain provides greater sensitivity in detecting brain responses than modeling the full pain block duration^46^. In particular, onset-evoked responses provide a robust marker of nociceptive input and are less confounded by habituation during sustained stimulation^47,48^. Condition onsets and durations for each condition were modeled according to the experimental design. A high-pass filter with a 128 s cutoff was applied to remove low-frequency drifts in the data. To account for head motion, as mentioned above, confound regressors were generated using FD values, with regressors censoring high-motion time points. The primary contrast of interest, pain ramp > rest, was used to assess brain activity during the early phase of pain perception.

To evaluate intervention-related changes in brain activation, subject-level difference maps were created in SPM using spm_imcalc, subtracting pre-intervention contrast images from post-intervention images. These maps were entered into a group-level analysis using a flexible factorial design in SPM12, with Group (EA vs. ML) as a factor. Age (centered at the overall mean) was included as a covariate of no interest, and changes in widespread pain were included as a covariate of interest. Clusters of activation were identified based on group differences in the relationship between changes in widespread pain and brain activation during the pressure-pain ramp versus rest condition. To focus the analysis on brain tissue, threshold masking was applied using the FSL MNI152 template to exclude non-brain regions. Voxel-wise inference was performed at *p* < 0.001 (uncorrected), with cluster-level family-wise error (FWE) correction at *p* < 0.05 to adjust for multiple comparisons. This analysis was designed to identify regions where nociceptive-evoked activation was specifically associated with changes in widespread pain, as our hypothesis was grounded in behavioral findings linking PPTol with widespread pain.

### Generalized psychophysiological interaction (gPPI) analysis

A Generalized Psychophysiological Interaction (gPPI) analysis was conducted using the CONN toolbox (v.20.b) to examine task modulated FC during pressure-pain stimulation. This approach was chosen because gPPI allows for the examination of task-dependent changes in connectivity across multiple conditions, providing a more comprehensive understanding of context-specific neural interactions compared to traditional correlation-based FC measures^49^. Six regions of interest (ROIs) within the insula were selected as seeds: the left and right anterior, middle, and posterior insula^50^ (radius = 6 mm) due to their established role in pain processing and their known aberrant FC in FM patients, which has been linked to altered pain perception^51^. Additionally, activation clusters in the primary somatosensory cortex, posterior midcingulate cortex, and precuneus, resulting from the activation analysis were included as seeds to capture task-specific brain regions involved in pain processing (Table 1).

**Table 1:** Regions of interest (ROIs) used for gPPI analysis

Seed ROI	Number of voxels (k)	MNI coordinates (x,y,z)
Left anterior insula (L aIC)	123	−32, 16, 6
Left middle insula (L mIC)	123	−38, 2, 8
Left posterior insula (L pIC)	136	−39, −15, 11
Right anterior insula (R aIC)	123	32, 16, 6
Right middle insula (R mIC)	123	38, 2, 8
Right posterior insula (R pIC)	112	39, −15, 8
Left somatosensory cortex (L S1)	75	−8, −38, 68
Right somatosensory cortex (R S1)	438	20, −34, 66
Left posterior midcingulate cortex (pMCC)	17	−14, −26, 38
Left Precuneus	223	−8, −58, 48

Each region of interest (ROI) in the table is identified by its anatomical label and hemisphere (L = left, R = right), along with the number of voxels included in the ROI mask and its Montreal Neurological Institute (MNI) coordinates (x, y, z).

Similar to the factorial structure used for the activation analysis, second-level analyses were modeled in CONN’s GLM framework with Group (EA vs. ML) as a factor, age (centered at the overall mean) as a covariate of no interest, and changes in widespread pain as a covariate of interest. Four conditions were modeled for both pre- and post-treatment sessions: rest, green fixation cross, pain ramp, and pain plateau. The primary contrast of interest was (pain ramp > rest) post-treatment minus (pain ramp > rest) pre-treatment, designed to assess related treatment-changes in FC during the pain ramp condition. Statistical analyses were performed using a voxel-wise threshold of *p* < 0.001 (uncorrected), with cluster-level family-wise error (FWE) correction at *p* < 0.05 to adjust for multiple comparisons.

### Mediation analyses

Mediation analyses were conducted to investigate the neural mechanisms linking changes in PPTol and widespread pain in the EA group. Specifically, we examined whether brain activation and FC measures from the gPPI analysis served as mediators in this relationship.

Two models were tested using SPSS’s PROCESS macro^52^ to evaluate mediation and serial mediation pathways. In the first model (Model 4), we tested a simple mediation framework where the independent variable (IV) was the change in PPTol following treatment, the dependent variable (DV) was the change in widespread pain, and the mediator was brain activation in pain-processing regions. This model assessed whether the relationship between improved nociceptive pain tolerance and reduced nociplastic pain was explained by activation changes in activated brain areas.

The second model (Model 6) applied a serial mediation framework incorporating two mediators in sequence: brain activation (M1) and FC between the activation cluster and other brain regions (M2). This model tested a theoretical pathway in which increased somatosensory input from EA leads to heightened primary somatosensory cortex (S1) activation (M1), which then enhances its connectivity with regions like the anterior insula (M2), ultimately resulting in a reduction in widespread pain.

All mediation analyses were conducted using bias-corrected bootstrapping with 5000 bootstrap samples to estimate indirect effects. Statistical significance was assessed with 95% confidence intervals, with indirect effects considered statistically significant if the confidence interval did not include zero. All mediation analyses were controlled for age.

## Results

### Increased pain tolerance to nociceptive-initiated stimuli following EA is associated with reduced nociplastic widespread pain

Treatment credibility was found to be equal across both the EA and ML groups. To examine changes in nociceptive and nociplastic pain in each group, we examined PPTol and widespread pain scores as PPTol reflects sensitivity to nociceptive pain stimuli, while widespread pain is a clinical marker of nociplastic pain (see Methods). Although neither the EA (widespread pain: pre = 5.37, post = 5.16; PPTol: pre = 3.79, post =  3.66) nor mock laser (ML; sham; widespread pain: pre = 5.00, post = 4.72; PPTol: pre =  3.50, post = 3.26) group showed statistically significant post-pre changes in PPTol or widespread pain, there was substantial inter-individual variability in change scores.

**Fig. 2:**
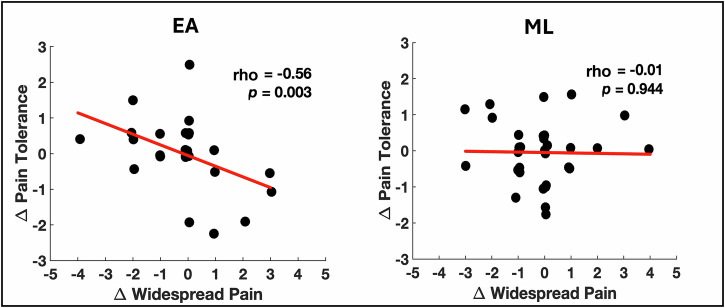
Changes in nociceptive-initiated pressure-pain tolerance and nociplastic widespread pain are related in EA but not ML. Scatter plots show the correlation between changes in widespread pain (x-axis; post-pre treatment) and changes in pressure-pain tolerance (y-axis; post-pre treatment in kgf/cm^2^) for the electroacupuncture (EA; *n* = 33) group (left) and mock laser (ML; *n* = 37) group (right). In the EA group, increased pain tolerance was significantly correlated with reduced widespread pain (rho = −0.56; *p* = 0.003), while no such relationship was observed in the ML group (rho = −0.01; *p* = 0.944). Two-sided Spearman correlation coefficients (rho) and p-values for each group are included in the plots as well. The red lines indicate the linear regression fits for each group.

In the EA group, increased PPTol was significantly correlated with reduced widespread pain (rho = −0.56, *p* = 0.003), while no such relationship was found in the ML group (rho = -0.01, *p* = 0.944; Fig. 2). In addition, there was a significant group difference in the strength of the correlation between changes in PPTol and widespread pain, with the relationship being significantly stronger in the EA group compared to the ML group (z = -2.0, *p* = 0.04).

### Brain activation in sensory and default mode network regions mediates the relationship between improved PPTol and reduced widespread pain in the EA group

**Fig. 3:**
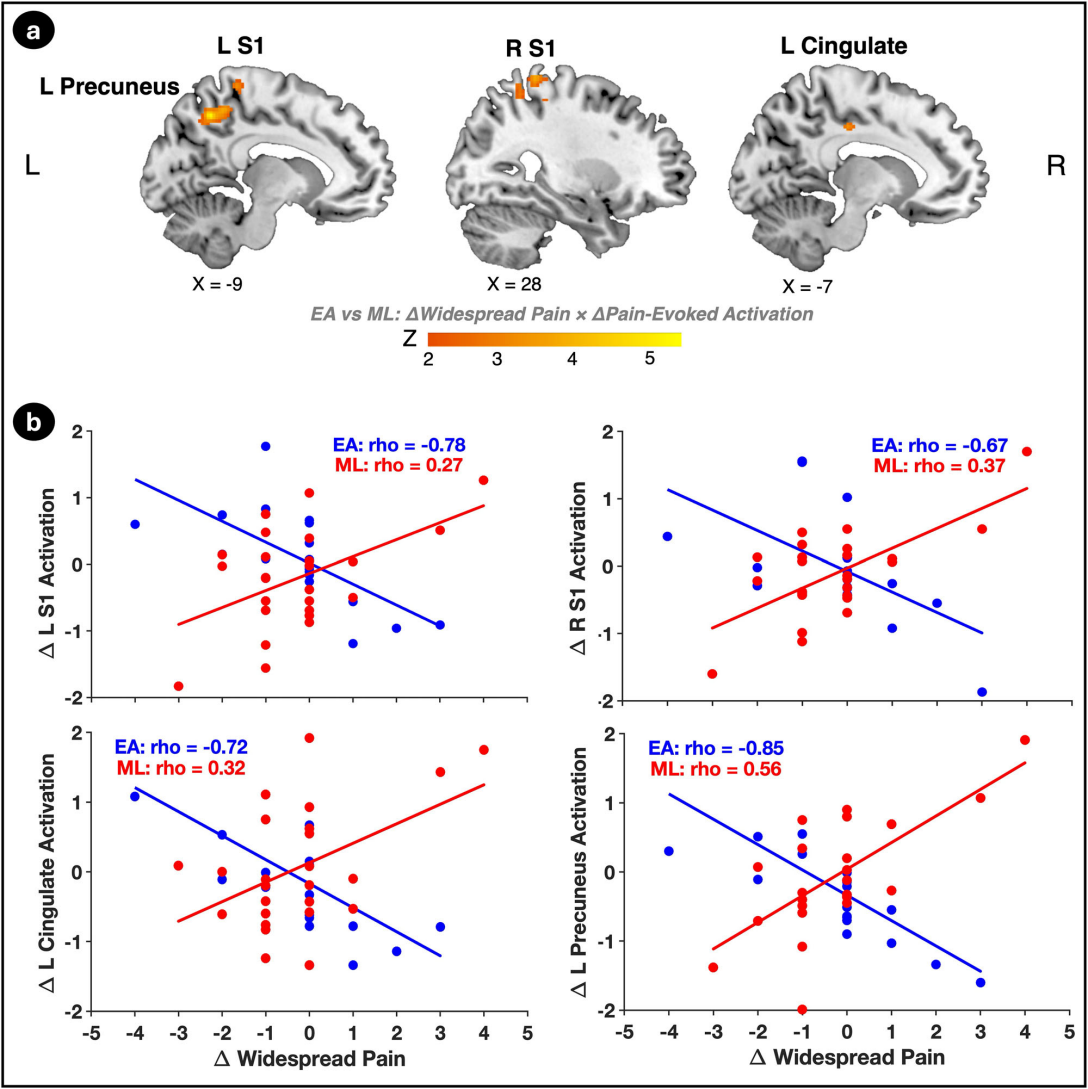
Associations between changes in brain activation and widespread pain differ between EA and ML. **a** Brain regions showing significant differences between electroacupuncture (EA; *n* = 19) and mock laser (ML; *n* = 25) in how activation changes during evoked pressure-pain relate to changes in widespread pain, including the left and right primary somatosensory cortices (L S1, R S1), left posterior midcingulate cortex (L pMCC), and left precuneus. The analysis was two-sided with a voxelwise threshold of *p* < 0.001 uncorrected and cluster-level family-wise error at *p* < 0.05. The color bar represents Z-scores. **b** Scatter plots display the correlation between changes in activation (% BOLD signal change, y-axis) and changes in widespread pain (# total body pain regions, x-axis) for the EA (blue) and ML group (red). In the EA group, greater increases in activation in these regions correlated with larger reductions in widespread pain, while the ML group showed positive correlations, particularly in the left precuneus. Spearman correlation coefficients (rho) are displayed for each group.

Results of a whole-brain activation analysis demonstrated significant group differences in the relationship between post- minus pre- treatment changes in brain activations during evoked pressure-pain and changes in widespread pain in four regions: the left (L) and right (R) S1, the left posterior midcingulate cortex (pMCC), and the left precuneus (Fig. 3; Table 2). In the EA group, greater activation in all four of these regions was associated with larger reductions in widespread pain. In contrast, the ML group showed positive correlations, particularly in the left precuneus, where reduced activation was linked to decreases in widespread pain (Fig. 3).

**Table 2:** Group-level differences between EA and ML treatments in associations between changes in brain activation and widespread pain

Cluster	Cluster Size (k)	ML > EA (T-statistic, *p*-value)	Peak Coordinates (x, y, z)
L S1	75	T = 4.32, *p* = 0.005	−8, −38, 68
R S1	438	T = 4.66, *p* < 0.001	20, −34, 66
L pMCC	17	T = 4.63, *p* < 0.001	−14, −26, 38
L precuneus	223	T = 4.67, *p* = 0.001	−8, −58, 48

The table presents group-level differences between electroacupuncture (EA) and mock laser (ML) where changes in brain activation were differentially correlated with reductions in widespread pain. Analyses were performed using a two-sided voxel-wise general linear model with a voxel-wise threshold of *p* < 0.001 (uncorrected) and a cluster-level family-wise error (FWE) correction of *p* < 0.05 for multiple comparisons. Significant clusters were found in the left and right primary somatosensory cortices (L S1: *p* = 0.005; R S1: *p* < 0.001), the left posterior mid-cingulate cortex (L pMCC: *p* < 0.001), and the left precuneus (L precuneus: *p* = 0.001). The table includes cluster sizes, T-statistics, significance values, and peak coordinates.

Furthermore, in the EA group, activations in three of these regions: right S1 (rho =  0.56, *p* = 0.013), left pMCC (rho = 0.59, *p* = 0.008), and left precuneus (rho = 0.50, *p* = 0.031)—were significantly correlated with improvements in PPTol, which was not observed in the ML group.

We hypothesized that brain activation would bridge the relationship between improved PPTol and reduced widespread pain, particularly in the EA group. Supporting this, mediation analyses showed that activations in all identified brain regions significantly mediated the link between increased PPTol and reduced widespread pain in the EA group (Table 3; example in Supplemental Fig. 1).

**Table 3:** Brain activations mediate the effects of increased pressure-pain tolerance on reductions in body pain regions following EA

Path		Mediator (M)			
		L S1	R S1	L pMCC	L Precuneus
Pain Tolerance (X) → Brain Activation (M)	Beta (SE)	0.41 (0.23)	0.37 (0.25)	0.62 (0.24)	0.43 (0.26)
	CI	[−0.05, 0.85]	[−0.12, 0.87]	[0.13, 1.10]	[−0.07, 0.94]
Brain Activation (M) → Widespread Pain (Y)	Beta (SE)	−1.25 (0.45)	−0.98 (0.43)	−0.99 (0.45)	−1.56 (0.38)
	CI	[−2.14, −0.35]	[−1.84, −0.13]	[−1.87, −0.11]	[−1.94, −0.40]
Pain Tolerance (X) → Widespread Pain (Y)	Beta (SE)	−0.42 (0.43)	−0.55 (0.45)	−0.31 (0.50)	−0.41 (0.42)
	CI	[−1.28, 0.44]	[−1.44, 0.34]	[−1.30, 0.69]	[−1.24, 0.41]
Pain Tolerance (X) → Brain Activation (M) →Widespread Pain (Y)	Beta (SE)	−0.50 (0.27)	−0.37 (0.29)	−0.61 (0.38)	−0.50 (0.34)
	CI	[−1.16, −0.09]	[−1.22, −0.04]	[−1.76, −0.20]	[−1.59, −0.12]

The table presents the direct and indirect effects of changes in brain activation in the left and right primary somatosensory cortices (L S1, R S1), left posterior midcingulate cortex (pMCC), and left precuneus, individually mediating the relationship between increased pain tolerance (independent variable; X) and reductions in widespread pain (dependent variable; Y) in the electroacupuncture (EA) group. The regression beta coefficients, standard errors (SE), and bootstrap confidence intervals (CI) [lower limit, upper limit] for both the effects are reported for each mediator (M).

### gPPI analyses reveal distinct connectivity patterns linked to widespread pain reduction in EA and ML groups

**Fig. 4:**
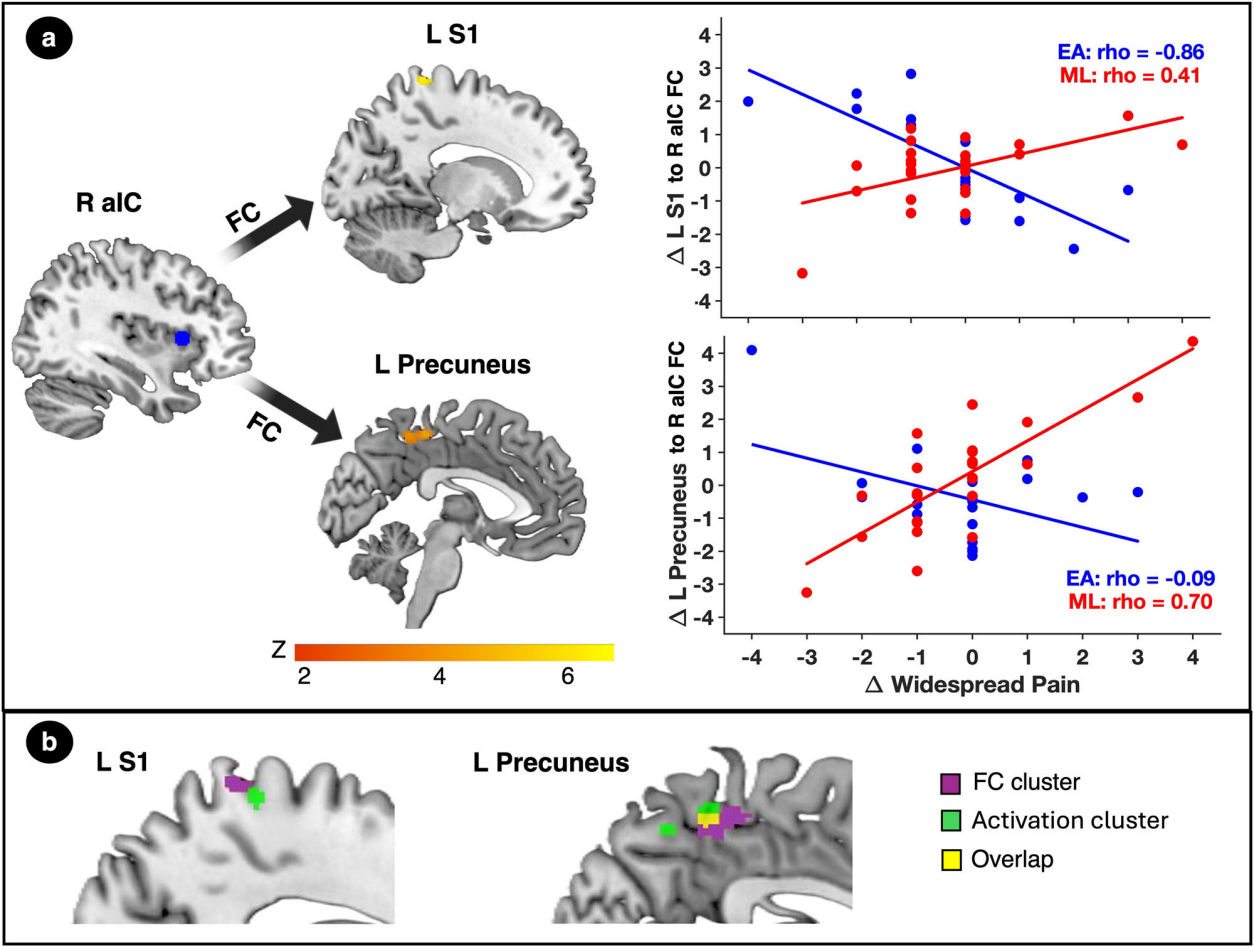
Changes in functional connectivity (FC) during pain and their relationship with widespread pain reduction. **a** Brain regions showing significant differences in the relation of FC changes during evoked pain with widespread pain between the electroacupuncture (EA; *n* = 19) group and mock laser (ML; *n *= 25) group, identified using group-level gPPI analysis (two-sided; voxel-level *p* < 0.001 uncorrected; cluster-level family-wise error correction at *p* < 0.05). Significant clusters included FC between the right anterior insula (R aIC) and left primary somatosensory cortex (L S1), as well as between R aIC and the left precuneus. Scatter plots depict correlations between changes in FC (y-axis) and changes in widespread pain (x-axis) for the EA group (blue) and ML group (red). Spearman correlation coefficients (rho) are reported for each group. **b** Spatial overlap between activation and FC clusters. Although the clusters in the L S1 are non-overlapping, they both fall within the somatotopic hand representation, indicating that they lie in close proximity and may reflect modulation of the same hand-related sensory area by EA. Overlapping activation and FC clusters were observed in the precuneus.

Group comparisons from the gPPI analysis revealed significant differences in the relationship between post-pre change scores in FC during evoked pressure-pain and widespread pain across the EA and ML groups. In the EA group, increases in FC between the right anterior insula seed (R aIC) and left S1 resulting cluster during the pain ramp relative to rest was strongly associated with greater reductions in widespread pain (rho = -0.86, *p* < 0.001; Fig. 4A). A similar association was observed between the left S1 activation cluster seed and a right S1 cluster (rho = -0.69, *p* = 0.001). In contrast, in the ML group, reductions in widespread pain were associated with decreased FC between the R aIC seed and a left precuneus cluster (rho = 0.70, *p* < 0.001; Fig. 4A). Notably, the left S1 clusters identified in the activation and FC analyses were spatially adjacent, while the precuneus clusters showed spatial overlap (Fig. 4B). Detailed information on significant clusters is provided in Supplemental Table 4.

### Modulation of pain tolerance and widespread pain through activation and subsequent connectivity in the EA group

Increased connectivity between L S1 and R aIC was not only correlated with reductions in widespread pain in the EA group but was also positively correlated with activation in L S1. We explored whether S1 activation may be contributing to the observed increase in its FC with the anterior insula, which was supported with the mediation analysis showing that FC between left S1 and R aIC mediated the relationship between S1 activation and reductions in widespread pain following treatment (Supplemental Table 5). Although L S1–R S1 FC was correlated with reductions in widespread pain, this pathway did not reach significance in the mediation analysis and therefore was not retained as a mediator.

**Fig. 5:**
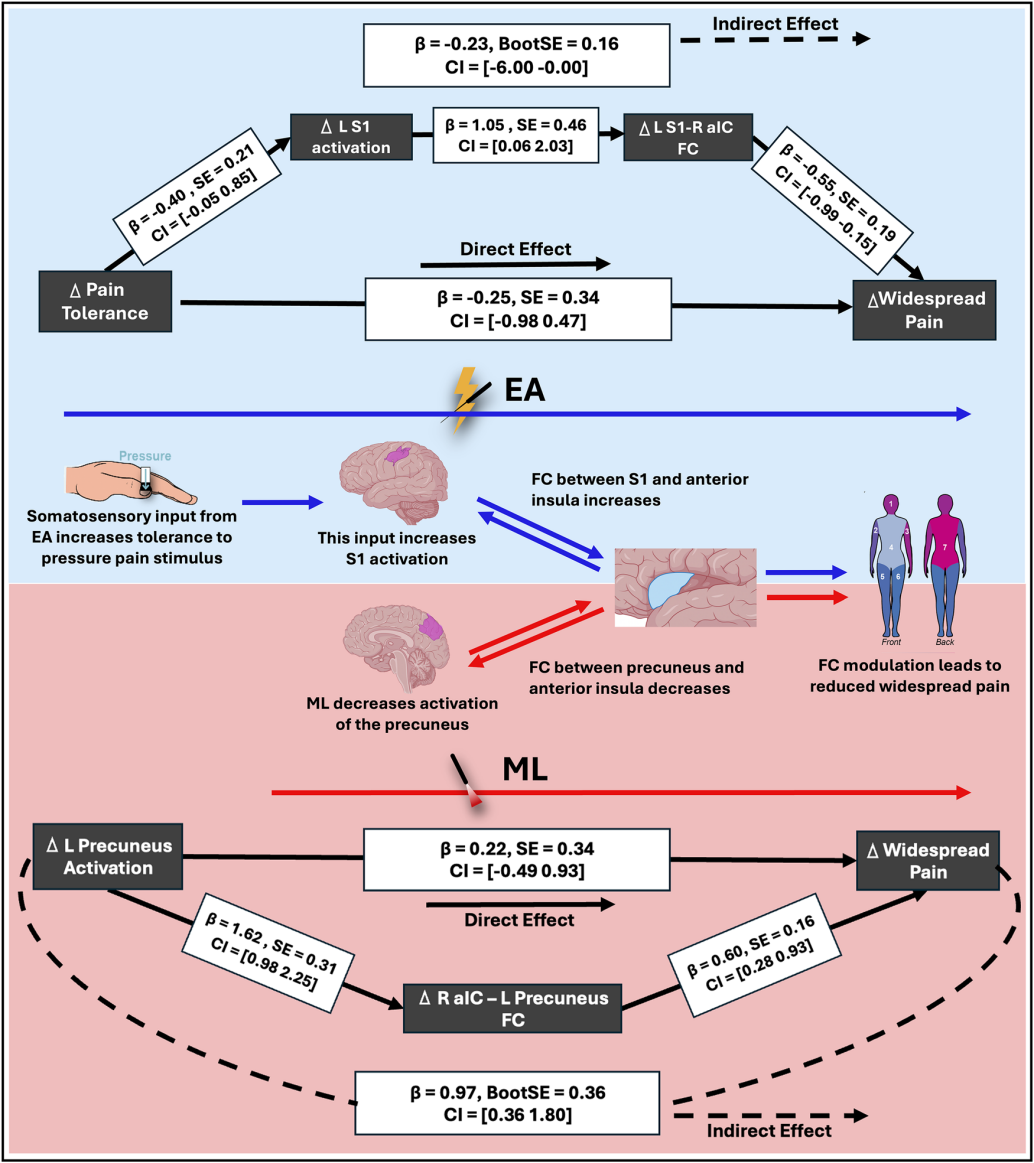
Mediation models for EA and ML examining brain pathways leading to widespread pain reduction. The top model highlighted in blue examines the indirect effects of left primary somatosensory cortex (L S1) activation and L S1 to right anterior insula (R aIC) FC in mediating the relationship between changes in pain tolerance and reductions in widespread pain in the electroacupuncture (EA; *n* = 19) group; while the bottom model highlighted in red examines the indirect effects of left precuneus activation and precuneus to R aIC FC in mediating the relationship between changes in pain tolerance and reductions in widespread pain in the mock laser (ML; *n* = 25) group. Mediation analyses were conducted using bias-corrected bootstrapping (5,000 resamples, two-sided). Solid lines represent direct effects, while dashed lines indicate indirect effects. Standardized beta coefficients (β), standard errors (SE), and confidence intervals (CI) are provided for each path. The middle panel illustrates the hypothesized mechanism in the EA (blue) and ML (red) groups.

A combined serial mediation model was constructed to test whether changes in PPTol were linked to reductions in widespread pain through sequential changes in S1 activation and connectivity: ΔPPTol (X) → ΔS1 activation (M1) → ΔS1–R aIC connectivity (M2) → Δwidespread pain (Y). Importantly, we modeled activation as preceding connectivity as regional activation is thought to initiate and shape subsequent network-level FC, rather than the other way around^28^. Both mediators significantly contributed to the pathway, indicating that increased PPTol leads to increased S1 activation, which then facilitates strengthened connectivity to the R aIC, culminating in reduced widespread pain (Fig. 5). Reversing the order of the mediators, placing FC before activation, did not yield significant effects.

In contrast, the ML group exhibited a different mechanism. In this group, reduced FC between the precuneus and the R aIC significantly mediated the relationship between decreased L precuneus activation and reduced widespread pain. (Supplemental Table 5).

## Discussion

Our findings highlight the distinction between nociceptive-initiated and nociplastic pain pathways, which often overlap but may require different treatment strategies. Nociceptive pain arises from peripheral injury or stimulation and is transmitted to the CNS via a “bottom-up” mechanism^2^. In contrast, nociplastic pain may be driven by both “bottom-up” and “top-down mechanisms”, the latter of which highlights corticospinal dysregulation^29^. This study examined the mechanistic neural pathway involved in altered pain processing during EA treatment for FM. Specifically, EA may reduce pain arising from both nociceptive-initiated pressure-pain and widespread nociplastic pain by engaging peripheral sensory input via acupuncture needle afference and then modulating central pain networks.

Our results may delineate a bottom-up pathway of EA that begins with improvements in PPTol suggesting that peripheral nociceptive input may initiate central changes. Although PPTol may be a composite measure that reflects both peripheral and central contributions to pain, it is fundamentally an evoked, nociceptive-initiated pain response driven by stimulation of peripheral A-delta fibers at the thumbnail bed. Acupuncture needle stimulation of the skin and deeper tissues (e.g., muscles, tendons, fascia), may thus, activate afferent somatosensory pathways that project to the brain’s S1, a region responsible for processing mechanical and tactile input from the body^53^. Such peripheral stimulation has been shown to increase activity in sensory regions such as S1, with greater activation associated with clinical pain reduction, as demonstrated in studies of carpal tunnel syndrome^54^. We also observed activation within the pMCC, a subregion of the cingulate strongly implicated in pain processing and motor–behavioral responses to nociception^55,56^, supporting its inclusion as part of the bottom-up circuitry engaged by EA.

Beyond local effects, S1 activation may promote stronger long-range connectivity between S1 and the aIC, a region involved in integrating sensory, emotional, and cognitive aspects of pain^57,58^. Within this pathway, S1 activation precedes S1–aIC connectivity, a pattern consistent with prior research indicating that regional activity can initiate and synchronize broader network interactions^59^. These functional changes may, in turn, contribute to a reduction in centrally maintained widespread pain, the hallmark of nociplastic pain in FM. Prior work^21^ supports this final stage of the pathway, showing that enhanced S1–aIC connectivity is associated with reductions in pain intensity, an effect mediated by elevated GABA levels in the insula. This suggests that shifts in neurotransmitter signaling within pain-modulatory regions may support the FC changes related to analgesia.

Together, these results indicate that EA may engage a sequential “bottom-up” mechanism: somatosensory input first influences local cortical activity in S1, which in turn modulates cortico-cortical FC with regions like the aIC, ultimately leading to reductions in widespread pain. This pathway, from peripheral stimulation to cortical activation, and from cortical activation to network-level integration, may be the mechanism through which EA exerts its analgesic effects. While these findings highlight a bottom-up pathway, they do not preclude the contribution of central influences, such as expectancy, contextual cues, or patient–practitioner interaction, that may also shape EA’s therapeutic effects^60^.

In contrast, ML, a sham intervention devoid of somatosensory signaling, likely operates more through a “top-down” mechanism on widespread nociplastic pain by targeting the precuneus, a key node of the DMN involved in introspection and self-referential thought, which are processes that may contribute to negative and maladaptive cognitive patterns in individuals suffering from chronic pain^61^. DMN hyperactivity has been consistently linked to heightened pain perception and emotional distress in chronic pain conditions, suggesting that deactivation of the DMN and reduction of DMN-insula connectivity may be potential mechanisms for attenuating widespread pain^62–65^. By reducing precuneus activation and its connectivity to the right anterior insula, ML may disrupt these maladaptive cognitive and emotional patterns, thereby contributing to pain relief. This finding aligns with prior research on placebo analgesia, which has similarly demonstrated changes in DMN activity and connectivity that modulate the cognitive-affective aspects of pain through expectation and belief^66,67^. Similar effects were observed in our recent study of cognitive behavioral therapy (CBT), a top-down pain-modulatory therapy, for chronic low back pain, where reductions in DMN–insula connectivity were strongly associated with pain relief^68^. Together, these findings suggest that DMN–insula decoupling may represent a shared mechanism underlying top-down analgesic effects across both psychological and expectation-driven interventions.

A major conceptual limitation of this study lies in the absence of gold-standard assays or experimental design to clearly distinguish between nociceptive and nociplastic pain mechanisms. While we interpret increased pressure-pain tolerance as a reflection of nociceptive-initiated pain, and widespread pain as a marker of nociplastic pain, these measures are not entirely distinct. Evoked pressure-pain tolerance, though peripherally driven, is also influenced by central modulation, and widespread pain, which is typically associated with central sensitization, may still be partially sustained by ongoing peripheral nociceptive input. This overlap complicates the attribution of specific neural changes to a single pain mechanism. Nevertheless, based on current scientific understanding and available tools, these measures represent the best available proxies for approximating nociceptive and nociplastic pain dimensions. In future studies, techniques such as microneurographic recordings from primary afferent fibers may help to more robustly segregate peripherally-driven from centrally-driven effects. In addition, although our mediation analyses were guided by theoretical rationale and empirical support, they should be interpreted as testing whether the data are consistent with hypothesized directional pathways linking clinical and brain changes, rather than as definitive evidence of causality. Another limitation is that, although inclusion of only female participants is consistent with prior FM neuroimaging studies, our findings may not generalize to males. Finally, because pain assessments were conducted a few days prior to the MRI sessions due to logistical constraints, this temporal separation between behavioral and imaging measures could introduce variability in the observed brain–behavior relationships.

The absence of statistically significant reductions in widespread pain and PPTol in the present sample may reflect variability in treatment response and limited statistical power given the modest sample size. This aligns with prior work showing that acupuncture in FM and other chronic pain conditions has produced mixed or nonsignificant results when compared with sham or placebo controls^69–71^, highlighting the ongoing challenge of disentangling specific treatment effects from nonspecific factors. One potential contributor to this variability is baseline sensory sensitivity, as prior studies have shown that individuals with heightened baseline sensory sensitivity (e.g., QST pressure pain) often respond less favorably to somatosensory-based treatments such as acupuncture or acupressure^30,72^. Consistent with this literature, in our sample, participants who exhibited decreases in PPTol after EA also tended to report greater widespread pain, suggesting that some individuals may worsen depending on their underlying pain profile.

Although some studies have reported modest or nonsignificant effects of EA, which may in part reflect differences in patient pain profiles and response variability, many other studies demonstrate that EA can significantly reduce widespread pain^73^ and pain severity^21^. Moreover, pain-tolerance seems to be implicated as baseline pressure-pain sensitivity has been shown to predict analgesic response to EA versus sham in FM^72^. Building on these findings, and acknowledging that heterogeneous pain profiles may differentially influence treatment benefit, the present study provides insights into the brain mechanistic pathway underlying the clinical effects of EA, showing how changes in PPTol and widespread pain are linked through specific brain circuits involved in sensory processing and integration.

Patients with FM exhibit significant variability in how much each of these pain mechanisms contribute to their symptoms. In some individuals, nociplastic pain may be more prominent, reflecting CNS dysfunction, whereas in others, nociceptive processes linked to peripheral abnormalities may play a larger role^2^. This variability highlights the importance of tailoring treatment approaches to the type of pain mechanisms in each patient. Treatments such as EA may be particularly beneficial for patients with mixed pain profiles, as they address both nociceptive and nociplastic components, whereas more “top-down” centrally acting treatments such as CBT may be more effective for patients with less nociceptive-initiated pain. Although we focus here on nociceptive and nociplastic mechanisms for clarity, other pain types, such as neuropathic pain^74^, may also play a role and should be considered in a comprehensive, individualized treatment strategy. Understanding the balance between these mechanisms in each patient is crucial for optimizing treatment outcomes and advancing personalized pain management strategies for pain disorders.

## Supplementary information

Supplemental Material

Description of Additional Supplementary files

Supplementary Data 1

## Data Availability

All source data underlying the figures and tables are provided in the Supplementary Data 1 (Excel file). Specifically, the source data for Figs. 2–5, Table 1, and Supplementary Tables 1–5 are included in this file. Additional de-identified behavioral and neuroimaging data (BIDS-formatted task fMRI, derived activation/connectivity measures, and clinical variables) are securely stored on University of California, Irvine (UCI) servers and are available from the corresponding author upon reasonable request.
