# Supplementary material for: Brain sensory network activity underlies reduced nociceptive initiated and nociplastic pain via acupuncture in fibromyalgia
Source: Commun Med (Lond). 2026 Jan 10;6:25. doi: 10.1038/s43856-025-01280-0 (PMC12808314; doi:10.1038/s43856-025-01280-0)
Supplement: Supplementary file 1 — Supplemental Material [file 43856_2025_1280_MOESM1_ESM.pdf]

Supplemental Section

Supplemental Fig. 1 Mediation model of the relationship between increased pressure-pain tolerance, greater S1 brain activation, and reduced widespread pain.

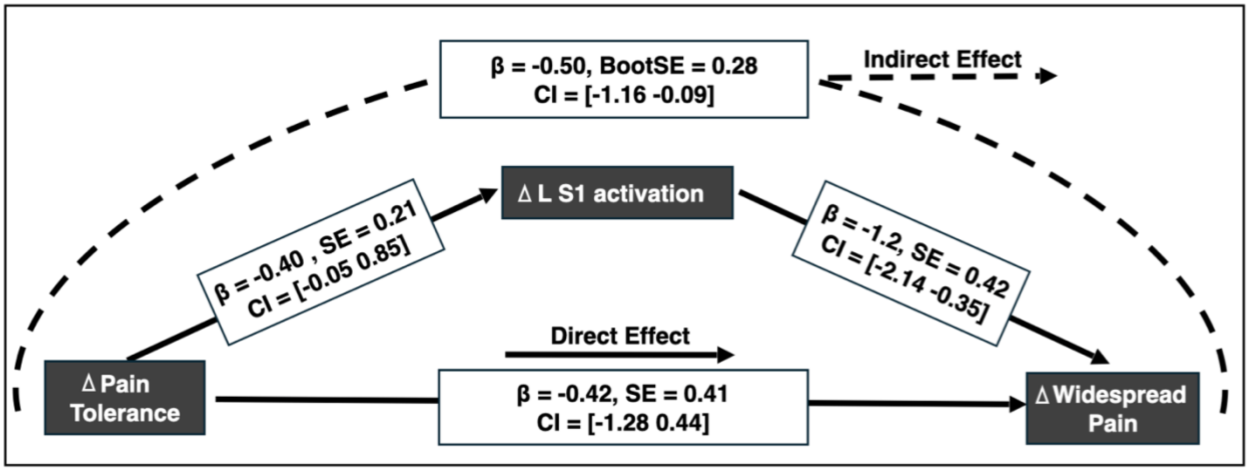

This mediation model shows that changes in L S1 activation significantly mediates the relationship between increased pain tolerance and reduced widespread pain. Solid lines represent direct effects, while the dashed line represents the indirect effect through L S1 activation. Standardized beta coefficients (β), standard errors (SE), and confidence intervals (CI) are reported for each path.

Supplemental Table 1. Participant demographics and clinical outcomes for full sample

	EA (N=33)		ML (N=37)		p-value		
	Pre-tx	Post-tx	Pre-tx	Post-tx	Time	Group	Group x Time
Age (years)	44.21±12.92		41.54±9.85		N/A	.339	N/A
Widespread Pain (Total # body pain regions)	5.52 ±1.56	5.18 ±1.61	5.03 ±1.36	4.76 ±1.62	.091	.164	.858
Pressure-Pain Tolerance (kgf/cm²)	3.88 ±1.48	3.88 ±1.23	3.66 ±1.61	3.62 ±1.43	.818	.514	.880
BPI Severity	5.21 ±1.47	4.07 ±1.91	4.93 ±1.33	4.47 ±1.62	<.001	.927	.042
BPI Interference	5.10 ±2.22	3.91 ±2.00	5.46 ±1.95	4.66 ±2.37	<.001	.231	.398
FM Survey Criteria	20.34 ±5.18	17.34 ±5.97	19.54 ±4.09	15.27 ±4.65	<.001	.108	.887

Participant demographics and clinical outcomes for the full sample of fibromyalgia participants (FM). Values represent group means ± standard deviation for the electroacupuncture (EA) and mock laser (ML) groups at pre- and post-treatment. P-values reflect results from two-sided repeated measures ANOVA testing for main effects of Time, Group, and the Group × Time interaction. BPI = Brief Pain Inventory. N/A = not applicable for between-group comparisons of baseline age.

Supplemental Table 2. Participant demographics and clinical outcomes for fMRI analysis

	EA (N=19)		ML (N=25)		*p*-value		
	Pre-tx	Post-tx	Pre-tx	Post-tx	Time	Group	Group x Time
Age (years)	43.58±14.58		41.08±11.22		N/A	.539	N/A
Widespread Pain (Total # body pain regions)	5.37 ±1.50	5.16 ±1.80	5.00 ±1.26	4.72 ±1.51	.283	.321	.878
Pressure-Pain Tolerance (kgf/cm²)	3.79 ±1.45	3.66 ±1.23	3.35 ±1.40	3.26 ±1.24	.329	.295	.869
BPI Severity	5.26 ±1.68	4.03 ±1.98	5.03 ±1.23	4.43 ±1.35	<.001	.842	.127
BPI Interference	5.32 ±2.38	4.01 ±1.83	5.42 ±2.16	4.74 ±2.36	.218	.498	.027
FM Survey Criteria	18.00 ±5.25	17.05 ±6.33	17.20 ±6.37	15.20 ±4.00	.027	.316	.417

Participant demographics and clinical outcomes for the fMRI analysis of fibromyalgia (FM) participants. Values represent group means ± standard deviation for the electroacupuncture (EA) and mock laser (ML) groups at pre- and post-treatment. P-values reflect results from two-sided repeated measures ANOVA testing for main effects of Time, Group, and the Group × Time interaction. BPI = Brief Pain Inventory. N/A = not applicable for between-group comparisons of baseline age.

Supplemental Table 3. Durations of fMRI Evoked-Pain Block Design Task

Condition	Duration (seconds)
Rest	10
Anticipation	4
Pain	10
Rest	12
Anticipation	8
Pain	10
Rest	20
Anticipation	6
Pain	10
Rest	14
Anticipation	10
Pain	10
Rest	10
Anticipation	4
Pain	10
Rest	12
Anticipation	10
Pain	10
Rest	14

Each row represents the sequential timing of rest, anticipation, and pain conditions within a single fMRI run. Pain blocks consisted of 10-second pressure stimuli (4-second ramp, 6-second plateau). Anticipation and rest blocks were jittered in duration to reduce predictability and improve model estimation.

Supplemental Table 4. Group-level differences in the relation between FC and widespread pain between EA and ML treatments.

Seed - Cluster	Cluster Size (k)	Contrast	*p*-value	Peak Coordinates of Cluster (x, y, z)
L S1 – R aIC	67	EA < ML	$p = 0.002$	-18, -40, +74
L S1 – R S1	52	EA < ML	$p = 0.005$	+28, -30, +48
L precuneus – R aIC	184	ML > EA	$p < 0.001$	-4, -34, +54

Clusters reflect regions where changes in brain activation in association with reductions in widespread pain is significantly difference between the electroacupuncture (EA) and mock laser (ML) groups. Analyses were performed using a two-sided two sample t-test, with a voxel-wise threshold of $p < 0.001$ (uncorrected) and cluster-level FWE correction of $p < 0.05$ to control for multiple comparisons. Significant clusters were identified for the following pairs: L S1–R aIC ($p = 0.002$), L S1–R S1 ($p = 0.005$), and L precuneus–R aIC ($p < 0.001$) The table includes cluster size, contrast, peak coordinates, and statistical significance.

Supplemental Table 5. Mediation Effects of FC on the Relationship Between Changes in Brain Activations During Pressure-pain and Reductions in Nociplastic Widespread Pain

Path		Group: EA<br><br>X: L S1 activation<br>M: L S1 to R aIC FC	Group: ML<br><br>X: L precuneus activation<br>M: L precuneus to R aIC FC
Path a: Brain Activation (X) → FC (M)	Beta (SE)	1.18 (0.41)	1.62 (0.31)
	Confidence Interval	[0.30, 2.05]	[0.98, 2.25]
Path b: FC (M) → Widespread Pain (Y)	Beta (SE)	-0.57 (0.18)	0.60 (0.16)
	Confidence Interval	[-0.96, -0.18]	[0.28, 0.93]
Path c: Brain Activation (X) → Widespread Pain (Y)	Beta (SE)	-0.76 (0.38)	0.22 (0.34)
	Confidence Interval	[-1.56, 0.05]	[-0.49, 0.93]
Path c′: Brain Activation (X) → Widespread Pain(Y) after controlling for FC (M)	Beta (SE)	-0.67 (0.30)	0.97 (0.36)
	Confidence Interval	[-1.36, -0.20]	[0.34, 1.78]

Mediation analysis results showing functional connectivity (FC) as a mediator (M) in the relationship between changes in brain activation (independent variable; X) and changes in widespread pain (dependent variable; Y) for EA and ML groups separately. In the EA group, increased left S1 activation (X) influences reductions in widespread pain (Y) through FC between the left S1 and right anterior insula (L S1 – R aIC FC). In the ML group, decreased left precuneus activation (X) influences reductions in widespread pain (Y) through reduced FC between the left precuneus and right anterior insula (L precuneus – R aIC FC). The table presents the beta coefficients, standard errors (SE), and confidence intervals (CI) for both direct and indirect effects. The direct effect represents the direct relationship between activation and changes in widespread pain, while the indirect effect reflects the mediated pathway through FC.
